# Case Report: Heterotopic pregnancy after adenomyosis surgery: a rare case highlighting diagnostic pitfalls and clinical insights

**DOI:** 10.3389/fmed.2025.1606074

**Published:** 2025-06-25

**Authors:** Qingqing Zhu, Shun Cao, Qi Wang, Jing Xu, Hongjie Hu

**Affiliations:** ^1^Department of Radiology, Sir Run Run Shaw Hospital, Zhejiang University School of Medicine, Hangzhou, China; ^2^Department of Pathology, Sir Run Run Shaw Hospital, Zhejiang University School of Medicine, Hangzhou, China

**Keywords:** heterotopic pregnancy, intramural pregnancy, adenomyosis, ultrasound, magnetic resonance imaging

## Abstract

We present an extremely rare heterotopic pregnancy (HP) in a 33-year-old patient with multiple adenomyosis surgeries and bilateral salpingectomy, who conceived via assisted reproductive technology (ART). According to a review of the literature spanning two decades, concurrent intrauterine and extrauterine pregnancies are uncommon, especially in the case of intramural ectopic gestations. On day 31 post-embryo transfer, only an intrauterine pregnancy (IUP) was observed by ultrasound. However, early vaginal bleeding and high-risk factors prompted further ultrasound, revealing an intramural ectopic pregnancy. Though MRI initially misdiagnosed the lesion, reevaluation led to the correct diagnosis. Surgical removal of the ectopic pregnancy was performed while preserving the IUP, which progressed uneventfully. In the late second trimester, follow-up MRI confirmed an intact posterior uterine myometrium, ruling out uterine rupture and resolving lingering concerns. This case illustrates a progression from incomplete ultrasound assessment to an initial misinterpretation of MRI. Ultimately, complementary imaging was vital for accurately diagnosing and managing the intramural ectopic pregnancy, while safeguarding the intrauterine pregnancy by confirming uterine wall integrity later on. Highlighting the complexity of HP in a patient with adenomyosis conceived via ART, it underscores the importance of multiple imaging techniques for early diagnosis and ongoing monitoring in high-risk scenarios. These findings guide clinical strategies and emphasize the critical role of accurate imaging in protecting both maternal and fetal wellbeing.

## 1 Introduction

Heterotopic pregnancy (HP) refers to the coexistence of intrauterine and ectopic pregnancies. Rare in natural conception, it becomes more likely with assisted reproductive technologies (ARTs) (1). Ectopic pregnancy rates rise from approximately 1 in 30,000 in natural conception (2) to 1 in 900 after ovulation induction, and 1 in 100 with ART (3). Maternal mortality for HP is approximately 5 per 1,000,000 (4).

Most ectopic pregnancies occur in the fallopian tube (1), but non-tubal varieties (cornual, ovarian, cesarean scar, cervical, intramural, and abdominal) also occur. Intramural pregnancy, involving implantation entirely within the myometrium without connection to the endometrial cavity or fallopian tube, is extremely rare, particularly when coexisting with an intrauterine gestation. Risk factors include prior uterine interventions (e.g., myomectomy, tubal surgery, or curettage), ART, and adenomyosis. Early diagnosis can be challenging due to variable presentations, and surgical intervention is usually required.

In this report, we describe a patient with adenomyosis, three prior surgeries, and bilateral salpingectomy, who underwent two ART cycles. The first cycle ended in an anembryonic miscarriage; after the second transfer of two embryos, she was diagnosed with a heterotopic pregnancy consisting of concurrent intrauterine and intramural ectopic gestations, the latter eventually arresting. Initially, MRI misinterpreted the intramural lesion as cystic adenomyosis, but subsequent reevaluation established the correct diagnosis. MRI then proved vital for continued monitoring. This case highlights the complementary roles of MRI and ultrasound in managing high-risk pregnancies and offers insights for more effective future monitoring.

## 2 Case report

**Figure 1:**
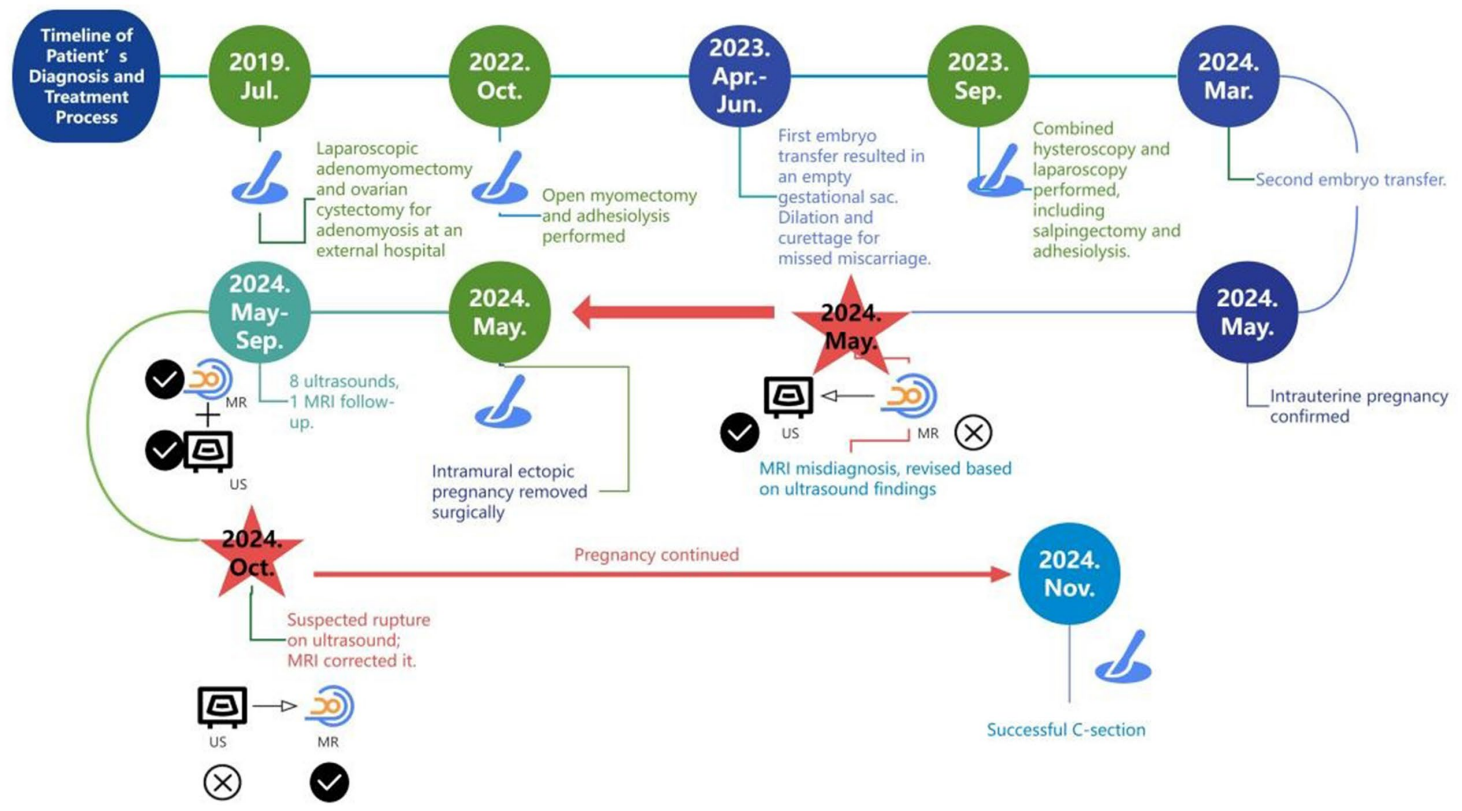
Timeline of the patient’s diagnosis and treatment process.

We present a 33-year-old woman with a history of three adenomyosis surgeries and bilateral salpingectomy, who underwent two frozen embryo transfers: the first failed, and the second resulted in a heterotopic pregnancy (comprising intrauterine and intramural components). Confirming the intramural component and monitoring the uterine wall throughout pregnancy proved diagnostically challenging (Figure 1).

The patient had a 10-year history of dysmenorrhea, which was managed with diclofenac. In July 2019, she underwent laparoscopic adenomyomectomy, left ovarian cystectomy, right mesosalpinx cystectomy, and bowel adhesiolysis. By 2022, her symptoms had worsened, leading to goserelin treatment on 28th September and open myomectomy on 7th October. The excised mass measured 60 × 60 × 45 mm, extending beyond half the myometrium; both adnexa were densely adherent to the posterior uterus. The rAFS score was 88.

After a failed embryo transfer in May 2023 and subsequent dilation and curettage (D&C) in June, she underwent combined laparoscopy and hysteroscopy in September, including bilateral salpingectomy, adhesiolysis, hysteroscopic lesion resection and biopsy, and laparoscopic coagulation of endometriosis. Dense adhesions formed a sealed pelvic cavity, and both tubes were rigid and narrowed, with a 30 × 20 mm hydrosalpinx on the left. Methylene blue showed no patency; the left tubal score was 21/24, and the rAFS score was 22.

**Figure 2:**
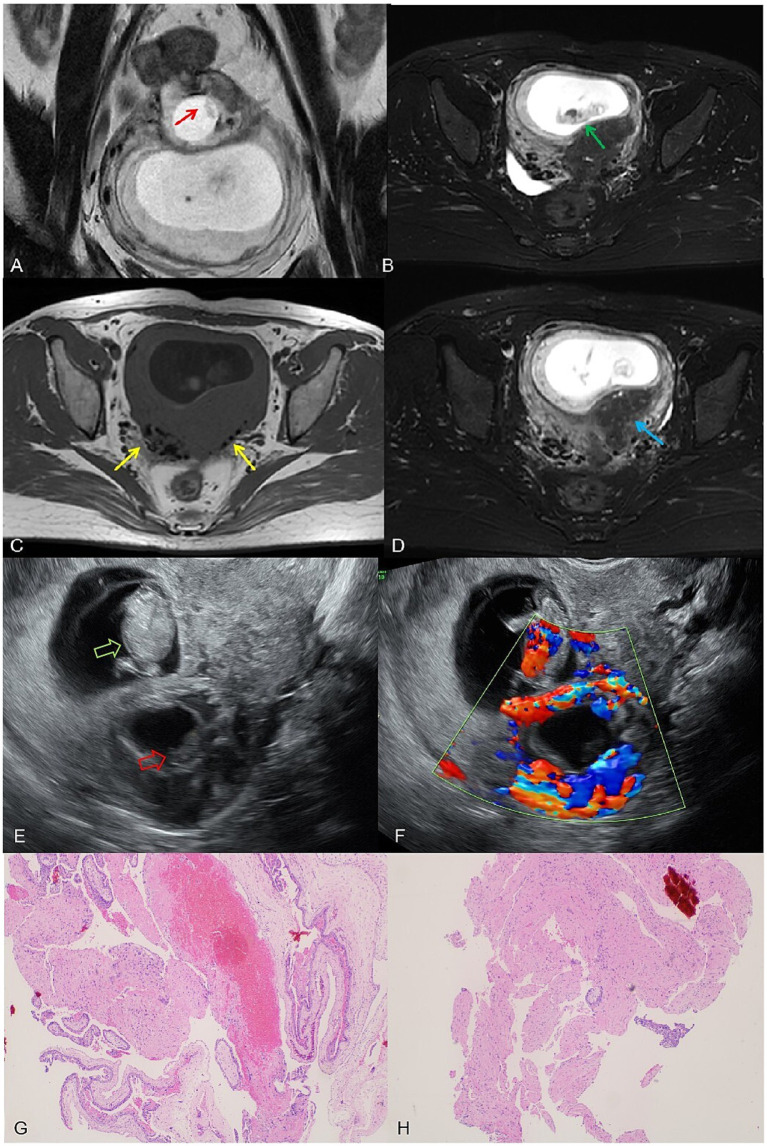
**(A)** A complete embryonic bud, measuring 8.9 mm, visible within the intramural gestational sac on the small T2 FSE sequence (red arrow). **(B)** A normal intrauterine pregnancy with a fetus measuring a crown-rump length of 34 mm on the T2-fs-FSE sequence (yellow arrow). **(C)** T1WI shows black speckled hypointense signals, considering postoperative changes (red arrow). **(D)** T2-FSE-SPAIR reveals residual adenomyosis with visible small cysts (yellow arrow). **(E)** Ultrasound image shows the intrauterine pregnancy fetus (green hollow arrow) and the intramural pregnancy fetus (red hollow arrow). **(F)** Color Doppler ultrasound image: cardiac activity detected in the intrauterine fetus, but absent in the intramural fetus; ring-shaped blood flow signals can be observed around the intramural gestational sac. **(G,H)** Postoperative pathological slides showing chorionic tissue (hematoxylin and eosin stain, magnification ×40).

On 14 March 2024, the patient underwent another embryo transfer. At 13th April (31 days post-transfer), ultrasound confirmed an intrauterine pregnancy (gestational sac 24.4 × 22.2 × 12.9 mm, embryo 7.2 mm) with slight bleeding. At 10 weeks (day 69 post-transfer), a 1.5 T MRI (United Imaging uMR560) confirmed an intrauterine pregnancy (gestational sac 62 × 41 × 70 mm, CRL 34 mm) but initially misidentified a 23 × 22.7 × 21 mm intramural pregnancy as cystic adenomyosis. Routine T2-FSE sequences obscured the intramural embryo, but a smaller-field T2-FSE revealed an 8.9 mm embryo in the intramural sac. A 3D T1 axial sequence partially depicted the embryo despite moderate signal limitations (Figure 2).

On the same day, transvaginal ultrasound showed an enlarged intrauterine sac (38.9 × 57.6 × 65 mm) with an embryo (CRL 38.9 mm) and cardiac activity. A smaller posterior myometrial lesion (23.7 × 18.9 mm) contained a 10.7 mm embryo-like structure without a yolk sac or cardiac activity, which was attached to the serosal layer with minimal myometrium and abundant blood flow. A 43.6 × 34.4 × 54.3 mm fluid-filled cystic area was noted near the right ovary. These findings led to a multidisciplinary MRI review, which confirmed intramural ectopic pregnancy. MRI also revealed adenomyosis, minor cystic changes in the lower uterine segment, and postoperative scarring (low-signal areas). The cervix measured 37 mm, showing stromal thickening likely due to pregnancy hormones. The patient underwent exploratory laparotomy under combined spinal-epidural anesthesia. A 5 × 4 cm intramural mass was found on the posterior uterine wall, densely adhered to the surrounding tissues. After careful adhesiolysis, the ectopic gestational tissue was excised from the myometrium, and the uterus was repaired in two layers with absorbable sutures. Both fallopian tubes were absent due to prior bilateral salpingectomy. Intraoperative blood loss was approximately 200 mL. The patient had an uneventful recovery and was discharged in stable condition.

**Figure 3:**
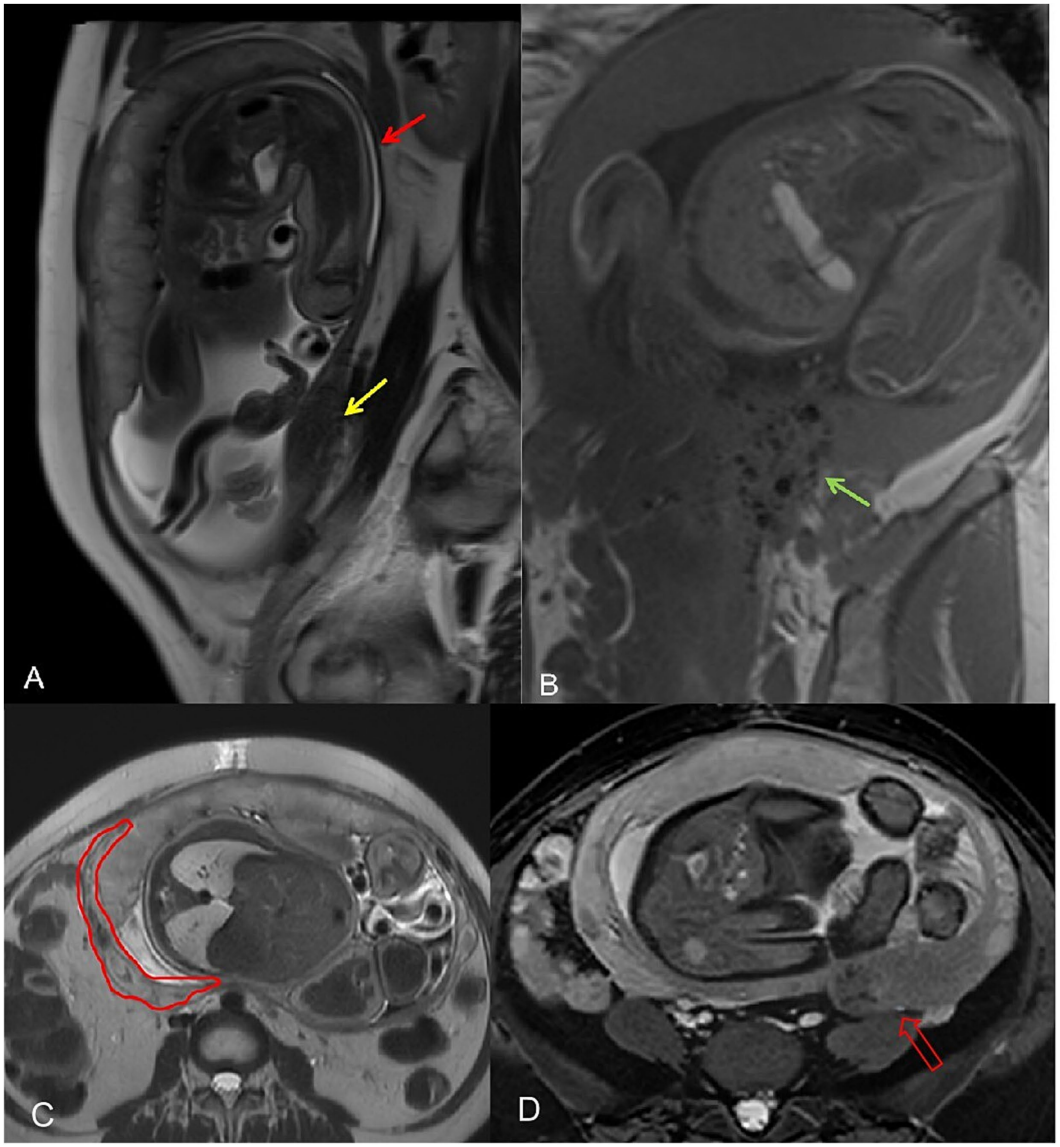
**(A)** The thinnest area of the myometrium in the posterior uterine walled arrow. A hypointense area is observed in the myometrium of the left posterior wall, considered to be an adenomyosis lesion (yellow arrow). **(B)** Postoperative speckled hypointense changes (green arrow). **(C)** The red outline indicates thickened intramural vessels, which were mistakenly interpreted as myometrial rupture on ultrasound. **(D)** The red hollow arrow points to the adenomyosis lesion area and the surrounding black speckled postoperative changes.

From 27 May to 30 September 2024, the patient had nine ultrasounds as part of prenatal care, including cervical length assessment and uterine scar surveillance. By 30th September, the posterior uterine wall’s thinnest area had measured 3 mm. On 11th October (32 weeks), MRI showed that the myometrium was thinned to 2 mm but remained intact, and a 50 × 16.3 × 67 mm low-signal region in the left posterior wall. The cervix was 27.4 mm, the anterior placenta was normal, and fetal measurements included a BPD of 81 mm and an HC of 293.9 mm (Figures 3A,B).

On 28th October (36 weeks), ultrasound revealed a hypoechoic zone between the posterior uterine serosa and myometrium, which suggested an intramyometrial blood sinus or partial rupture. The same-day MRI (United Imaging 1.5 T uMR680) confirmed a dilated blood sinus with no rupture. A 70 × 22 × 90.9 mm low-signal area in the left posterior uterine wall contained several cystic foci, likely adenomyosis with postoperative changes (Figures 3C,D).

**Figure 4:**
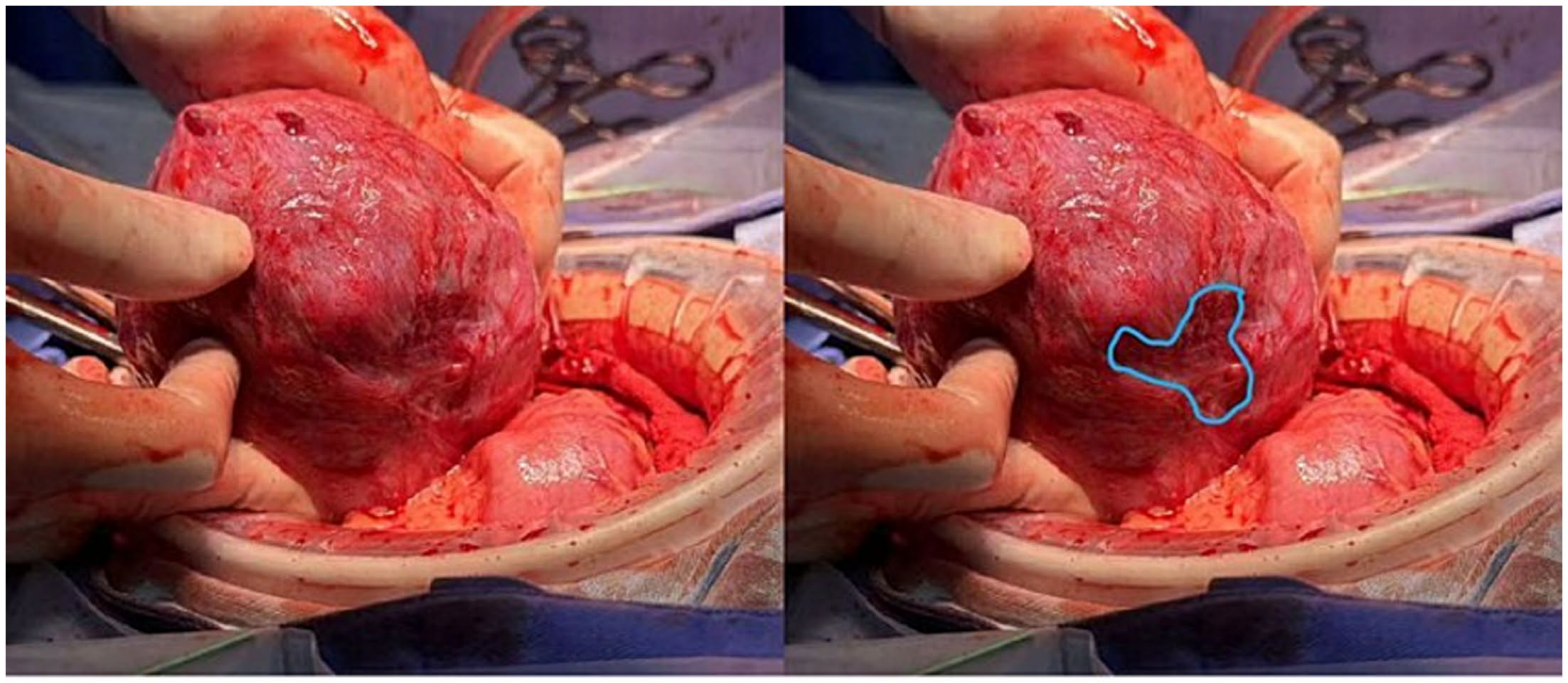
Intraoperative view of the uterus after delivery of the fetus and placenta. The posterior myometrium was routinely examined. A well-defined area of myometrial thinning was delineated (outlined in blue), with no signs of uterine wall rupture observed.

On 1st November (35 weeks), the patient underwent a cesarean section, delivering a healthy female infant (Apgar scores 10 and 10 at 1 and 5 min, respectively). The placenta was delivered intact, and localized thinning was noted in the posterior midline uterine wall (Figure 4). Estimated blood loss was 400 mL.

## 3 Discussion

Ectopic pregnancy (EP) remains a significant cause of morbidity and mortality in early pregnancy. Its signs and symptoms can be subtle; up to 50% of patients may initially be asymptomatic (5–7). The two primary symptoms—abdominal pain and vaginal bleeding—can also occur in intrauterine pregnancies (IUPs) or threatened miscarriages, often complicating diagnostic accuracy. Even when both symptoms are present, an EP diagnosis may still be overlooked (8). Although the primary function of *β*-hCG is to confirm pregnancy and evaluate early gestational development, it does not indicate the implantation site if there is a concurrent viable IUP (1). Ultrasound serves as the most important diagnostic tool. However, identifying an intrauterine embryo on ultrasound may give a false sense of security regarding a normal pregnancy, thereby overshadowing the potential for an ectopic pregnancy (9). In a study by Jeon et al. (7), only 16% of asymptomatic heterotopic pregnancies were diagnosed early via transvaginal ultrasound in IVF patients. This increases the risk of rupture, life-threatening hemorrhage, and possible hypovolemic shock. Therefore, early and accurate identification of ectopic pregnancy is crucial for improving both maternal outcomes and preserving fertility (10). Alongside imaging, monitoring pregnancy risks, increasing the frequency of early pregnancy ultrasound and closely observing clinical symptoms are all essential.

The patient in this case had multiple risk factors for EP: adenomyosis, a history of multiple surgeries (including bilateral salpingectomy), and two cycles of assisted reproductive technology (ART) (11–13). Adenomyosis not only indicates structural changes in the uterus and compromised endometrial integrity but is also associated with implantation failure and an increased risk of ectopic pregnancy (14). Although surgical interventions may alleviate symptoms and improve fertility, they can also weaken the uterine wall, potentially creating myometrial defects that predispose patients to intramural or other forms of ectopic pregnancy. ART further elevates the risk of multiple gestations and ectopic implantation; women undergoing ART have an approximately 8-fold higher risk of EP (15). Additionally, mechanical manipulations during embryo transfer and uterine contractions induced by luteal support may encourage ectopic embryo implantation. Recognizing these risk factors is crucial, as they align with known risk factors for both heterotopic pregnancy (HP) and EP, including pelvic inflammatory disease, a history of ectopic pregnancy, prior tubal surgeries, endometriosis, the use of intrauterine devices (IUDs), advanced maternal age, smoking, and infertility treated with ART (6, 16, 17).

In this case, frequent imaging during the early pregnancy phase enabled the timely detection of the intramural pregnancy without severe complications. Careful and thorough imaging is vital for identifying EP at an early stage. Locating an intrauterine pregnancy does not exclude the possibility of an ectopic pregnancy, and vice versa (18). The patient’s IUP was initially diagnosed on ultrasound at 31 days post-embryo transfer. Early vaginal bleeding and multiple risk factors prompted closer ultrasound surveillance, leading to the diagnosis of HP at day 54 post-transfer. Thus, detailed clinical history and imaging evaluation play equally important roles (17–20). Although ultrasound remains the frontline imaging modality for pregnancy, magnetic resonance imaging (MRI) can further delineate complex anatomy and guide treatment planning (17). MRI is increasingly utilized in complicated pregnancies, as it offers high-resolution anatomical detail. Intramural pregnancy typically appears on MRI as a cystic lesion within the myometrium, often surrounded by a rim of hypointense myometrium; an embryo or a gestational sac-like structure may be identifiable within the lesion.

In this case, the initial MRI failed to detect the intramural pregnancy, largely due to the lack of baseline imaging prior to conception and partial volume effects that obscured the embryo (Supplementary Data S2: The Mechanism of Misdiagnosis of MRI). This underscores the importance of high-resolution, multi-sequence imaging. In later pregnancy, MRI can also compensate for ultrasound’s limitations, particularly in assessing uterine wall integrity, placental location, and lesion progression. Here, MRI was instrumental in ruling out uterine rupture and informing subsequent pregnancy management. Ultrasound excels at early pregnancy detection and real-time observation of the embryo; however, MRI provides crucial supplemental information in complex scenarios (20). Indeed, ultrasound correctly identified HP in this case, while MRI initially misdiagnosed the lesion as cystic. Contributing factors to the misdiagnosis included an incomplete clinical history, inadequate review of ultrasound findings, the small size of the failed embryo (approximately 10 mm in length and 3 mm in thickness), and the relative rarity of early pregnancy MRI, which may limit radiologists’ experience with diagnosing blastocyst morphology. Nevertheless, MRI proved invaluable later on by compensating for ultrasound “blind spots.” At 33 weeks, MRI confirmed intact myometrial continuity, enabling her pregnancy to continue and alleviating the patient’s anxiety. At 35 weeks, when ultrasound suggested possible partial myometrial rupture but was hindered by fetal positioning, MRI showed that the uterine wall vascularity was increased but intact, thereby ruling out rupture. Three days later, the patient underwent an elective cesarean delivery.

Throughout this case, imaging was pivotal in diagnosis, guiding treatment decisions, and follow-up. Heterotopic pregnancy (HP) significantly differs from standard EP in that preserving the IUP is paramount. Methotrexate is contraindicated, making treatment more complex (21, 22). Once an EP is diagnosed, prompt intervention is ideal to prevent rupture and emerging complications. For unruptured ectopic gestations, local injection therapies (e.g., KCl or hypertonic glucose, excluding methotrexate) or surgical intervention can be considered. Surgical removal of an ectopic pregnancy is generally safe and does not elevate the risk of fetal loss in the concurrent IUP (7). Moreover, surgical treatment allows direct removal of the ectopic mass while preserving the intrauterine pregnancy.

In summary, a comprehensive imaging strategy—combining ultrasound and MRI—is crucial for diagnosing, managing, and following up on heterotopic pregnancies. By integrating clinical risk factors with detailed imaging findings, clinicians can optimize patient outcomes and mitigate the potential complications associated with HP. To translate the insights from this case into practical guidance for clinical decision-making, especially in the context of assisted reproductive technology (ART) and complex uterine anatomy, we propose the following key takeaways for clinicians:

1. Ultrasound remains the first-line imaging modality for early pregnancy evaluation due to its accessibility, safety, and real-time capability in detecting intrauterine or ectopic gestations.
2. Upgrade to MRI when:
  1. Ultrasound findings are inconclusive or ambiguous, particularly in patients with uterine abnormalities such as adenomyosis or surgical scars.
  2. There is a discrepancy between clinical presentation and imaging findings, or suspicion of rare ectopic types (e.g., interstitial and intramural).
  3. Detailed anatomical evaluation of uterine wall integrity, lesion morphology, or placental location is needed in late gestation.
3. Continue with ultrasound when:
  1. Pregnancy findings are clear and consistent with the clinical scenario.
  2. Serial monitoring of IUP development or postoperative recovery is necessary.
4. In ART patients with complex uterine anatomy:
  1. Maintain a high index of suspicion for HP, even with confirmed IUP.
  2. Recognize adenomyosis, prior surgery, and ART as key risk factors for ectopic gestation.
  3. Combine clinical history, *β*-hCG trends, and serial imaging for accurate diagnosis.
  4. Acknowledge that early MRI may miss subtle gestational structures if protocols are not tailored for pregnancy.
5. Apply a phased imaging strategy:
  1. Early pregnancy: Use ultrasound for localization and viability.
  2. Mid-to-late pregnancy: Use MRI when ultrasound is compromised or high-risk features emerge.
  3. Postpartum/follow-up: Use ultrasound routinely; reserve MRI for deep or complex lesion evaluation.
6. In cases of diagnostic uncertainty:
  1. Repeat imaging with adjusted parameters.
  2. Document interpretive limitations.
  3. Consider surgical evaluation if imaging is non-diagnostic but clinical concern persists.

From the patient’s perspective, initial diagnostic uncertainty and misdiagnosis were sources of anxiety and distress. However, once a definitive diagnosis was made and effective treatment was administered, the patient expressed a sense of relief and satisfaction. Her experience highlights the psychological impact of delayed diagnosis and underscores the importance of clear doctor–patient communication and the complementary use of various imaging modalities when dealing with rare or atypical diseases.

## 4 Conclusion

This case report presents a rare instance of concurrent intrauterine and ectopic pregnancy following IVF-ET in a patient with a history of adenomyosis and multiple pelvic surgeries. The diagnostic and management challenges underscore the critical importance of multimodal imaging in high-risk pregnancies. Future research should aim to optimize imaging protocols and develop personalized management strategies for patients with adenomyosis and postoperative conditions, thereby improving pregnancy outcomes and enhancing maternal and neonatal safety.

## Data Availability

The raw data supporting the conclusions of this article will be made available by the authors, without undue reservation.
