# Supplementary material for: Case Report: Heterotopic pregnancy after adenomyosis surgery: a rare case highlighting diagnostic pitfalls and clinical insights
Source: Front Med (Lausanne). 2025 Jun 25;12:1606074. doi: 10.3389/fmed.2025.1606074 (PMC12237884; doi:10.3389/fmed.2025.1606074)
Supplement: Supplementary file 1 [file Data_Sheet_1.pdf]

Supplementary Material

1.Supplementary Data

1.1 Supplementary Data S1: Operation Record

Hospital: The Sir Run Run Shaw Hospital of Zhejiang University School of Medicine
Patient ID: [Hidden]
Date of Admission: 2024-05-07 21:40
Date of Operation: 2024-05-10
Ward Number: [Hidden]
Gender: Female
Age: 33 years
Department: Gynecology
Primary Surgeon: [Hidden]
Assistant Surgeon: [Hidden]
Anesthesiologist: [Hidden]
Anesthesia Method: Combined spinal-epidural anesthesia (CSEA)
Surgical Duration: 100 minutes
Blood Loss: 200 ml

Preoperative Diagnosis:
- Cesarean scar pregnancy (to be confirmed)

Pregnancy Status: Pregnant

Postoperative Diagnosis:
- Heterotopic pregnancy
- Intrauterine pregnancy with scar pregnancy (intramural)

Surgical Name:
- Laparotomy for resection of ectopic pregnancy and clearance of uterine intramural pregnancy

Surgical Findings:
Dense adhesion between uterus and bowel due to previous cesarean scar. Dense adhesion on both sides of the uterus and pelvic sidewalls. Partial adhesion between the uterus and the bowel and abdominal wall. Uterine posterior wall with a 5×4 cm mass, surface similar to myometrium, adhered to small intestine and omentum. After incision, chocolate-like contents observed, myometrial wall was thin and inner cavity contained gestational tissue about 2×2 cm in size. Gestational tissue removed and basal layer cleaned.

Surgical Procedure:

①After successful anesthesia, routine disinfection and draping were performed.

②A 6 cm lower abdominal transverse incision was made; electric scalpel used to cut through subcutaneous tissue and linea alba.

③The bladder peritoneum was separated and pushed down.

④The uterus was exposed and lifted out of the incision.

⑤Posterior uterine wall was cut open to expose mass; gestational tissue was found and cleared; 1-0 absorbable sutures used to close the uterine wound in two layers.

⑥Repeated irrigation with warm saline, surface covered with Interceed to prevent adhesions, abdomen closed layer by layer.

⑦Surgery was uneventful; patient transferred to PACU postoperatively.

Implants: None
Specimens: Intramural gestational tissue

Transfusion: None
RBC: 0 unit | Platelets: 0 unit | Plasma: 0 ml | Whole Blood: 0 ml

Pathology: Pending

Chief/Assistant Signature: [Hidden]
Attending Physician: [Hidden]
Surgery Start Time: 2024-05-10 11:37
Surgery End Time: 2024-05-11 12:13

1.2 Supplementary Data S2: The Mechanism of Misdiagnosis of MRI

①Partial Volume Effect

This effect occurs when MRI voxels encompass signals from multiple tissue types, leading to signal averaging and reduced contrast between adjacent structures. This can obscure small or subtle lesions, especially when their size approaches the voxel dimensions.

In this case, the intrauterine fetus is consistent with gestational age, with a crown-rump length of approximately 34 mm. However, the intramural pregnancy has ceased development, with the embryonic bud measuring only 8.9 mm in length and 3 mm in width. Most MRI sequences used have a slice thickness of 4 mm and an interslice gap of 0.8 mm. Only the small-field T2-weighted transverse sequence has a

thinner slice thickness of 3 mm and a 0.3 mm gap. Due to the relatively small size of the lesion compared to the slice thickness, the partial volume effect likely contributed to the lesion being overlooked by the radiologist.

②Sequence Resolution (Spatial and Temporal)

Spatial and temporal resolution are critical in detecting small or subtle lesions. A small gestational sac embedded in the myometrium can mimic a T2-hyperintense cyst, which is commonly seen in cystic adenomyosis. Without high-resolution imaging, these entities may be indistinguishable.

In this case, the small intramural gestational sac was misinterpreted as a benign T2-hyperintense lesion, likely due to limited spatial resolution. Routine pelvic MRI protocols often omit 3D isotropic sequences such as T2-weighted SPACE, which provide thinner slices and the ability to perform multiplanar reconstructions (MPR).

③Lack of Motion Suppression

Uterine peristalsis and bowel movement can significantly degrade pelvic MRI image quality. When motion suppression techniques—such as breath-holding instructions or administration of antiperistaltic agents (e.g., hyoscine butylbromide, glucagon)—are not employed, image blurring may occur.

In this case, no breath-holding was performed during scanning, and no antispasmodic agents were administered. The lesion was closely adjacent to bowel loops, which further contributed to motion artifacts and image blurring. This significantly reduced the visibility of lesion margins and may have caused it to mimic a T2-hyperintense adenomyotic cyst rather than being recognized as an intramural pregnancy. When fine embryonic structures are not clearly depicted due to motion degradation, the risk of misdiagnosis increases substantially.

④Absence of Targeted Protocol for Pregnancy

Standard pelvic MRI protocols designed for evaluating gynecologic conditions or pelvic pain often do not include sequences specifically tailored to detect early gestational sacs, especially those in ectopic or atypical locations such as the myometrium.

Moreover, contrast agents are contraindicated during pregnancy, limiting the ability of MRI to visualize the rich vascular supply of an intramural gestational sac. This further complicates differentiation from non-vascular T2-hyperintense lesions, such as cystic adenomyosis. In this regard, ultrasound—which can demonstrate vascular

flow via Doppler imaging—offers a distinct advantage for early pregnancy assessment.

2. Supplementary Tables

2.1 Supplementary Table S1：Imaging Features Comparison: Cystic Adenomyosis vs Intramural Pregnancy

Feature	Cystic Adenomyosis	Intramural Pregnancy
**Location**	Within the myometrium, usually near junctional zone	Within the myometrium, away from endometrial cavity
**Morphology**	Ill-defined, multiple small or solitary cysts	Well-defined, round or oval gestational sac
**Signal on T2-weighted MRI**	High signal cysts, surrounding low-signal myometrium	High signal sac with possibly hypointense rim
**Signal on T1-weighted MRI**	May be hyperintense if hemorrhagic	Usually low signal (unless hemorrhage present)
**Wall characteristics**	Thin, irregular walls, may be ill-defined	Thickened, decidualized wall may be seen
**Surrounding myometrium**	Diffusely thickened with indistinct JZ	Normal or focally disrupted myometrium
**Associated findings**	May have multiple adenomyotic foci elsewhere	No other adenomyotic foci; possible peritrophoblastic flow on Doppler
**Contrast enhancement (if done)**	Minimal or peripheral enhancement	Ring enhancement of gestational sac (if viable)
**DWI characteristics**	Typically no restricted diffusion	Restricted diffusion may be present
**Clinical correlation**	Chronic pelvic pain, dysmenorrhea	Amenorrhea, positive β-hCG, abnormal bleeding

2.2 Supplementary Table S2:MRI Technical Parameters for Three Imaging Sessions

MRI System	Sequence Type	Slice Thickness (mm)	matrix size	TR (ms)	TE (ms)	FOV (mm)	Gadolinium Used
**uMR560**	T1-fse(tra)	4.0	304 × 85	750	8.76	360 × 360	No
	T2-fse-spair(sag)	4.0	320 × 80	4310	88.38	280 × 280	
	T2-fse-spair(tra)	4.0	320 × 80	5550	98.2	360 × 280	
	DWI(b=50, 800 s/mm²)	4.0	128 × 100	3306	74	360 × 260	
	Small-T2-fse(tra)	3.0	256 × 90	4200	104.2	180 × 180	
**uMR680**	T1-quick3d	4.0	256 × 75	4.6	1.66	320 × 320	
	T2-ssfse（tra,cor,sag）	4.0	256 × 75	1500	96.56	300 × 300	
	Bssfp	4.0	336 × 85	5.19	2.35	360 × 360	
	DWI(b=50, 800 s/mm²)	4.0	128 × 100	5626	93.6	380 × 320	
**Vida**	T2-haste(axi,cor,sag)	3.0	256 × 162	882	97	280× 218.8	
	T1-flash-2d	4.0	160×90	220	2.46	320×240	
	DWI (b= 0, 600 s/mm²)	4.0	50×80	4700	77	158×102.7	
